# Expression changes in DNA repair enzymes and mitochondrial DNA damage in aging rat lens

**Published:** 2010-08-27

**Authors:** Yi Zhang, Lu Zhang, Lan Zhang, Jie Bai, HongYan Ge, Ping Liu

**Affiliations:** 1 Eye hospital, The First Affiliated Hospital, Harbin Medical University, Harbin, China; 2 Cardiovascular medicine, The Fourth Affiliated Hospital, Harbin Medical University, Harbin, China

## Abstract

**Purpose:**

To determine if there is increased mitochondrial DNA (mtDNA) and nuclear DNA (nDNA) damage with age in the lenses of rats. We also explored the immunolocalization of 8-oxoguanine DNA glycosylase 1 (OGG1) and AP endonuclease 1 (APE1) in the lens and studied three of the predominant base excision repair (BER) enzymes: OGG1, APE1, and DNA polymerase γ (Polγ).

**Methods:**

The methods used by this study include the selection of twenty-six male Wistar rats in each group (2 months old and 26 months old) and fourteen male Wistar rats in the 16 months old group. The total DNA of lenses were isolated and the DNA genome was amplified by a long extension–polymerase chain reaction (LX-PCR). We examined mtDNA and nDNA damage with a quantitative polymerase chain reaction (QPCR) assay that was combined with EvaGreen. We also studied the gene expression of mRNA and protein in these key BER enzymes with real time-polymerase chain reaction (RT–PCR) and western blot analysis.

**Results:**

There was an increase in oxidative DNA damage, which exists primarily in the mtDNA. The amount of 8-hydroxy-2’-deoxy-guanosine (8-OHdG) in DNA was significantly increased with age. Our experiments demonstrated that the gene expression of mRNA and protein in these key BER enzymes decreased with age. OGG1 and APE1 were localized by immunohistochemistry within lens epithelial cells (LECs) and superficial fiber cells.

**Conclusions:**

The gene expression of mRNA and protein in these key BER enzymes decreased with age, which caused a decrease in the repairing capability of the mtDNA and the accumulation of mtDNA damage. The increased mtDNA damage and decreased expression of BER enzymes may cause a “vicious cycle” of oxidative stress that contributes to the accumulation of mtDNA mutations and age-related cataract pathogenesis.

## Introduction

Age-related cataract is a leading cause of blindness worldwide and is a multifactorial eye disease [1]. Oxidative damage resulting from reactive oxygen species (ROS) is considered to be a major risk factor in the pathogenesis of both age-related and diabetic cataract [2]. ROS is mostly generated within the mitochondria in lens epithelium and the superficial fiber cells, which are highly reactive and can damage macromolecules in living cells, such as lipids, proteins, and nucleic acids, causing mutagenesis and cell death [3-6]. Mitochondrial DNA (mtDNA) is highly susceptible to the damage produced by ROS because of its close proximity to ROS generation through the respiratory chain and its paucity of protective histones [7-9]. Abnormal mitochondrial behavior resulting from mtDNA damage induced by oxidative stress has long been recognized as an important mediator of cell apoptosis. Moreover, the apoptosis of lens epithelial cells (LECs) may plays an important role in the pathogenesis of cataracts [10,11].

Aging is an inevitable biologic process that is associated with declining biochemical and physiologic function of the cell. The “mitochondrial theory of aging” suggests that aging results from declining mitochondrial function, due to high loads of damage and mutation in mtDNA. Oxidative damage to mtDNA has been implicated as a causative factor in a wide variety of degenerative diseases, in cancer, and in aging [12-15]. Under normal growth conditions, ROS leads to a low level of mtDNA and nuclear DNA (nDNA) damage, which is rapidly repaired, and most oxidative DNA lesions are repaired by the base excision repair (BER) pathway [7-9]. The BER pathway involves a highly coordinated process catalyzed by the sequential actions of DNA repair enzymes.

Many recent studies have focused on the role of mitochondria as mediators of oxidative damage in aging and diseases. Mitochondrial dysfunction, ROS formation, and oxidative damage of protein are associated with cataract formation, glaucoma, and retinal degeneration [16-19]. However, age-related damage to mtDNA in the lens has not been characterized in vivo. The purpose of the study presented here was to determine if there is an increased mtDNA and nDNA damage in the lens with age. We have characterized and compared the state of the mtDNA and nDNA in young and old rats’ lenses by quantitative polymerase chain reaction (QPCR) assay. Because DNA repair pathways are very important in protecting DNA against the deleterious effects of ROS, we also studied three of the predominant BER enzymes (8-oxoguanine DNA glycosylase 1 [OGG1], endonuclease 1 [APE1], and DNA polymerase γ [Polγ]) and explored the immunolocalization of OGG1 and APE1 in the lenses.

## Methods

### Animals

Twenty-six male Wistar rats in each group (2 months old and 26 months old) and fourteen male Wistar rats in the 16 months old group were provided by Animal Laboratories (Harbin Medical University, Harbin, China). Animals used for experiments were handled in strict accordance with the Association for Research in Vision Ophthalmology Statement on the Use of Animals in Ophthalmic and Vision Research.

### Long extension-polymerase chain reaction (LX-PCR)

LX-PCR was performed as previously described [20]. Genomic DNA in lens capsules with adherent LECs tissue from eight rat eyes in each group was isolated with a DNeasy Tissue Kit (W6501; Watson Bio, Shanghai, China). Quantitation of the purified genomic DNA, as well as that of PCR products, was performed fluorometrically using the EvaGreen dsDNA reagent (Biotium, Hayward, CA). EvaGreen is a novel DNA intercalating dye that is more stable and sensitive than SYBR Green I [21-24]. The assay, with a linear detection range of 0.2 to 1,000 ng, is extremely sensitive for dsDNA. LX-PCR was performed with the GeneAmp PCR system (Applied Biosystems, Foster City, CA), using long DNA polymerase enzyme (TAKARA,Dalian,China) which is designed to amplify target DNA sequences up to about 20 kb. The amounts of primers were 20 pmol. The four pairs of PCR primers employed in this study are given in Table 1. For amplification of a long fragment of mtDNA (13.4 kb), the standard thermocycler program included initial denaturation at 94 °C for 5 min, with 18 cycles of 94 °C for 30 s and 68 °C for 12.5 min, and with a final extension at 72 °C for 10 min. To amplify a long fragment of nDNA (12.5 kb), the thermocycler profile included initial denaturation at 94 °C for 5 min, and 28 cycles of 94 °C for 30 s and 68 °C for 12 min, with a final extension at 72 °C for 10 min. DNA damage was quantified by comparing the relative efficiency of amplification of large fragments of DNA (13.4 kb from mtDNA and 12.5 kb from nDNA) and normalizing this to the amplification of smaller (235 bp and 195 bp) fragments [20]. Total cellular DNA concentration was determined with EvaGreen dsDNA quantitation reagent. Each sample was run in triplicate, and each experiment was performed at least twice.

**Table 1:** Primers used for PPCR amplification.

**Long fragment of mtDNA (13.4 kB)**
5′-AAAATCCCCGCAAACAATGACCACCC-3′ Sense
5′-GGCAATTAAGAGTGGGATGGAGCCAA-3′ Antisense
**Short fragment of mtDNA (235 bp)**
5′-CCTCCCATTCATTATCGCCGCCCTTGC-3′ Sense
5′-GTCTGGGTCTCCTAGTAGGTCTGGGAA-3′ Antisense
**Long fragment of nDNA (12.5 kB) from the clusterin (TRPM-2) gene, accession number,**M64733
5′- AGACGGGTGAGACAGCTGCACCTTTTC-3′ Sense
5′-CGAGAGCATCAAGTGCAGGCATTAGAG-3′ Antisense
**Short fragment of nDNA (195 bp)**
5′- GGTGTACTTGAGCAGAGCGCTATAAAT-3′ Sense
5′- CACTTACCCACGGCAGCTCTCTAC-3′ Antisense

### Amplification of total, non-deleted and deleted mtDNA

Three non-overlapping regions of the mitochondrial genome were amplified by polymerase chain reaction (PCR) to evaluate the relative proportion of wild type (non-deleted, 369 bp), deleted mtDNA (348 bp), and total mtDNA (504 bp). Genomic DNA in lens capsules with adherent LECs tissue from eight rat eyes in each group was isolated with DNeasy Tissue Kit (W6501; Watson Bio). The three pairs of PCR primers employed in this study are given in Table 2. The total primer pair amplified a region of mtDNA outside the 4.8-kb common deletion, the wild-type primer pair amplified a region within the common deletion, and the deletion primer pair amplified a product only in the presence of the common deletion, which would bring the primer pairs within 500 bases [19]. The deletions predominantly occur between thermodynamically stable direct repeats, for which several mechanisms have been proposed. In rats, two 16-bp direct repeats flank a 4.8-kb region of mtDNA, referred to as the common or hot spot deletion, which includes six structural genes for subunits of complex I, IV, and ATPase, and five tRNAs [25]. Thermocycling included one cycle of 94 °C for 5 min, followed by 35 cycles of 94 °C denaturing for 15 s, 63 °C annealing for 45 s, and a 72 °C extension for 1 min. A final 72 °C extension for 5 min completed the PCR. Just as aforedescribed, the amount of PCR product was determined fluorometrically using the EvaGreen dsDNA quantitation reagent. Each sample was run in triplicate, and each experiment was performed at least twice.

**Table 2:** Primers for mtDNA amplification.

**Total mtDNA (504 bp)**
5′-CACACTCTCACTCGCATGAA-3′ Sense
5′-TCCTTCCAATCTAGTTGAGG-3′ Antisense
**Non-deleted mtDNA (369 bp)**
5′-ACTCCAACTCCATAATCTCC-3′ Sense
5′-TATTAGTGGGAGGAGTCAAG-3′ Antisense
**Deleted mtDNA (348 bp)**
5′-GGTCTACCAATTGTTGTGAC-3′ Sense
5′-TAGTGAGATAAGGAAGCCTG-3′ Antisense

### 8-hydroxy-2’-deoxy-guanosine ELISA

Competitive ELISA assays for 8-hydroxy-2’-deoxy-guanosine (8-OHdG) were performed according to the manufacture’s protocol (goat anti-rat 8-OHdG ELISA kit; CD-A55105; ADL, CA). Genomic DNA in lens from eight rat eyes in each group were isolated with gDNA Isolation Kit (W6501; Watson Bio). Standards and samples were incubated with biotinylated 8-OHdG in microtitration wells, which have been coated, with other 8-OHdG molecules with defined and unique epitope specificity. After incubation and washing, the wells are treated with streptavidin labeled with enzyme horseradish peroxidase (HRP). The wells are washed again, a TMB substrate solution is added to the wells, and color develops in proportion to the amount of 8-OHdG bound. The stop solution changes the color from blue to yellow, and the intensity of the color was measured at 450 nm. Standard 8-OHdG was assayed over a concentration range of 0 to 250 ng/ml in duplicates for each experiment. The average concentration of 8-OHdG per microgram of DNA for each group was calculated for each sample. Sample DNA assays were performed in duplicate. Controls without added DNA and appropriate blanks were also incorporated into experiments.

### Real time reverse transcriptase–polymerase chain reaction

The total RNA from the lens capsules with adherent LEC tissue was extracted by a RNA Isolation Mini kit/DNA-free (D7001; Watson Bio) according to the manufacturer’s instructions. RNA concentration and purity were determined on a spectrophotometer by calculating the ratio of optical density at wavelengths of 260 nm and 280 nm. One μg of RNA was reverse transcribed into cDNA using M-MuLV reverse transcriptase (TAKARA) according to manufacturer’s protocol using a total reaction of 20 μl. RT–PCR was performed using 1.25 μl of 20× concentrate EvaGreen for a final volume of 25 μl containing 50 ng of cDNA. For determination of the initial relative quantity of cDNA, samples were amplified with *OGG1*, *APE1*, *Polγ*, and glyceraldehyde-3-phosphate dehydrogenase (*GAPDH*) primers. The four pairs of PCR primers employed in this study are given in Table 3. Reactions were run on an Applied Biosystems Prism 7000 real time PCR machine (Applied Biosystems). The mixtures were initially denatured at 94 °C for 5 min. The PCR consisted of 40 cycles at the following conditions: denaturation at 94 °C for 30 s, annealing at 60 °C (for *OGG1*, *APE1*, and *Polγ*) for 30 s, and an extension period at 72 °C for 30 s. An internal standard was set up to normalize the relative gene expression level, and this standard was run with each different experiment. Melt curves analyses were performed for all genes and the specificity as well as integrity of the PCR products were confirmed by the presence of a single peak. Relative expression was calculated from the differences in cycle time of an internal standard (*GAPDH*) compared to the target mRNA. Duplicate CT values were analyzed in Microsoft Excel (Microsoft) using the comparative CT (2^-△△CT^) method (Applied Biosystems).

**Table 3:** Primers for RT–PCR.

***OGG1* primers**
5′- GACTCAGACCGAGGATCAGCTC-3′ Sense
5′- GCTATAGAGCTGAGTCAGGCTGAC-3′ Antisense
***APE1* primers**
5′- CAGATCAGAAAACGTCAGCCAG-3′Sense
5′- GGTCTCTTGGAGGCACAAGATG-3′Antisense
***Polγ* primers**
5′- GAAGAGCGTTACTCTTGGACCAG −3′Sense
5′- AACATTGTGCCCCACCACTAAC −3′Antisense
***GAPDH* primers**
5′-GTATTGGGCGCCTGGTCACC-3 ′ Sense
5′-CGCTCCTGGAAGATGGTGATGG-3′ Antisense

### Immunoblot analysis

Rat lens capsules with adherent LEC tissue were homogenized in chilled Trisbuffer (pH 6.0) containing 1% Triton X-100, phenylmethyl sulfonyl fluoride (10 mg/ml) and aprotinin (2 mg/ml). Proteins (50 μg) were separated by SDS–PAGE in 4% stacking and 10% separation gels. Proteins were then transferred to nitrocellulose filter (NC) membrane. After blocking with 10% BSA (BSA) overnight at 4 °C, each membrane was incubated for 2 h at room temperature with one antibody: an anti-rat OGG1 rabbit polyclonal antibody (1:800; Novus Biologicals, Littleton, CO), an anti-rat APE1 rabbit polyclonal antibody (1:700; Novus Biologicals), or an anti-rat Polγ goat polyclonal antibody (1:500; Santa Cruze Bio-technology Inc., San Diego, CA). After washing, the membrane incubated with OGG1 or APE1 rabbit polyclonal antibody was incubated for 1 h at room temperature with goat anti-rabbit IgG conjugated to horseradish peroxidase (1:700 Zsbio, Beijing, China), the membrane incubated with Polγ goat polyclonal antibody was incubated for 1 h at room temperature with rabbit anti-goat IgG conjugated to horseradish peroxidase (1:700; Zsbio). As an internal control, the membrane was incubated in a blocking buffer containing a 1:5,000 dilution of anti-rat GAPDH mouse monoclonal antibody (Santa Cruz Biotechnology Inc.) using the same conditions described above. The immunoblots were scanned and relative band density was determined using the Gel-Pro application (Media Cybernetics, Silver Spring, MD). The densities were normalized to GAPDH. Each experiment was performed a minimum of three times.

### Immunohistochemistry

The eyes were fixed overnight in 10% neutral buffered formalin. Paraffin was removed in xylene and a series of graded ethanol steps and rehydrated in PBS. The tissues were heated at 95 °C to 99 °C in citrate buffer (pH 6.0) for 20 min for antigen retrieval. The endogenous peroxidase activity was blocked by soaking in 0.03% H_2_O_2_ for 10 min. The sections were incubated overnight with a primary antibody at 4 °C, a rabbit anti-Rat APE1 antibody (1:200; Novus Biologicals), and a rabbit anti-Rat OGG1 antibody (1:300; Novus Biologicals). Control tissues were treated in the same way, but without antibody, to confirm that staining was specific to the antigen tested. After several washes, tissue sections were incubated with the secondary antibody, Polymerized HRP-Anti rabbit IgG (KIT-9901; Maxim. Bio, Fuzhou, China) for 1 h at room temperature. After washing with PBS, sections were treated for equal time in the DAB reagent and concurrently photographed.

### Statistics

For all experiments, data are reported as mean±SD as indicated, and a p value <0.05 was considered statistically significant. For the western blot assay, significant differences between groups were determined by an independent sample Student’s *t*-test (2-tailed) using SPSS version 15.0 software. For other assay, one-way ANOVA was used for testing statistical significances between groups of data.

## Results

### Long extension-quantitative polymerase chain reaction of mitochondrial DNA and nuclear DNA

**Figure 1:**
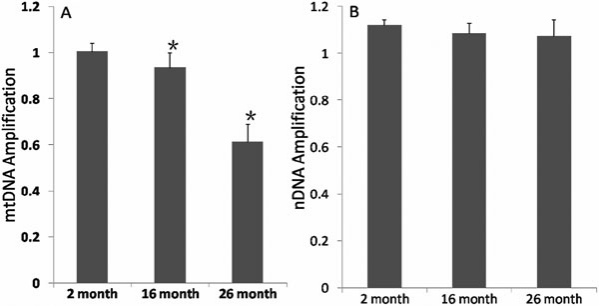
Increased mtDNA damage compared to nDNA in aged rat lens. Long fragments of mtDNA (13.4 kb) and nDNA (12.5 kb) from lens were measured. These data were normalized by the measured levels of the short fragment of mtDNA and nDNA obtained using the same DNA sample. **A**: there was a significant decrease in mtDNA amplification at 16 months (p=0.03, n=8) and at 26 months (p<0.01, n=8), compared with the 2 month group. There was a significantly increase at 26 months compared to 16 months group (p<0.01, n=8). **B**: there was no significant change in the relative amplification of nDNA at 16 months (p=0.149, n=8) and 26 months (p=0.06, n=8), compared with 2 month group. There was not a significantly change at 26 months (p=0.606, n=8) compared to 16 months group.

In our study, we used QPCR-based measurements to evaluate oxidative damage in nDNA and mtDNA. The approach is based on the principle that DNA lesions, including oxidative damage that cause strand breaks, base modification, and a basic sites, will block the progression of DNA polymerase and result in decreased amplification of the targets. The approach required small amounts of genomic DNA and was used to directly compare the damage of nDNA and mtDNA in the same sample [20]. The total input of genomic DNA was 15 ng for each PCR reaction. As shown in Figure 1A, the amplification of mtDNA decreased gradually in 16 month and 26 month old rats. There was a significant decrease in mtDNA amplification at 16 months (0.93±0.06, p=0.03, n=8) and at 26 months (0.61±0.08, p<0.01, n=8), compared with the 2 month group (1.01±0.04, n=8). There was a significantly increase at 26 months compared to 16 months group (p<0.01, n=8). In Figure 1B, it is shown that there was no significant change in the relative amplification of nDNA at 16 months (1.09±0.04, p=0.149, n=8) and 26 months (1.07±0.07, p=0.06, n=8), compared with 2 month group (1.12±0.02, n=8). There was not a significantly change at 26 months compared to 16 months group (p=0.606, n=8). The DNA concentration of the samples is calculated based on a DNA standard curve, plotting the fluorescence values on a Microsoft Office Excel (Microsoft, Redmond, WA) spreadsheet. A strong linear relationship (R^2^=0.994) was observed and this was reproducible. We have adopted EvaGreen as means to quantify DNA. These data showed there was increased mtDNA damage with age in the lens.

### Amplification of total, non-deleted and deleted mtDNA

**Figure 2:**
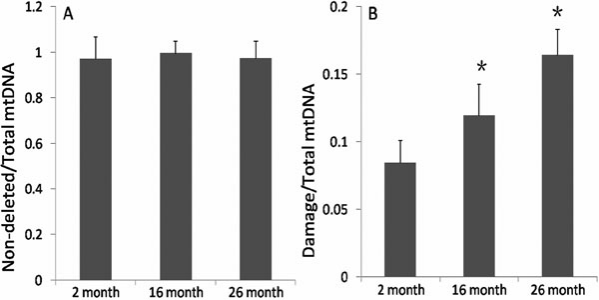
Increased levels of deleted mtDNA in aged rat lens. Measurements of levels of the PCR products of non-deleted (**A**) and deleted (**B**) mtDNA normalized by total mtDNA were done using the EvaGreen reagent. **A**: there were no significant change in non-deleted mtDNA at 16 months (p=0.51, n=8) and at 26 months (p=0.93, n=8), compared with the 2 month group. There was not a significantly change at 26 months compared to 16 months group (p=0.56, n=8). However, there was a significant increase in deleted mtDNA at 16 months (p=0.002, n=8) and at 26 months (p<0.001, n=8), compared with the 2 month group in **B**. There was a significantly increase at 26 months (p<0.01, n=8) compared to 16 months group.

Amplification products representing the wild-type, deleted and total mtDNA within homogenized tissue were detected using the same genomic DNA. The total input of genomic DNA was 15 ng for each PCR reaction. Deleted mtDNA was amplified from all groups by conventional PCR. As shown in Figure 2A, there were no significant change in non-deleted mtDNA at 16 months (1.00±0.05, p=0.51, n=8) and at 26 months (0.97±0.08, p=0.93, n=8), compared with the 2 month group (0.97±0.10, n=8). There was not a significantly change at 26 months compared to 16 months group (p=0.56, n=8). However, there was a significant increase in deleted mtDNA at 16 months (0.12±0.02, p=0.002, n=8) and at 26 months (0.16±0.02, p<0.001, n=8), compared with the 2 month group (0.08±0.02, n=8) in Figure 2B. There was a significantly increase at 26 months compared to 16 months group (p<0.01, n=8). These amplification data suggested that aging is strongly correlated with the accumulation of oxidative damage and mutation in mtDNA. However, a small proportion of mtDNA mutations could cause significant metabolic deficits, because the damage would be propagated as mitochondria and cells divide.

### 8-hydroxy-2’-deoxy-guanosine ELISA

**Figure 3:**
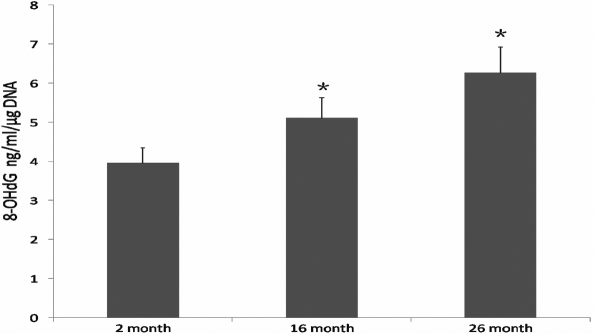
Increased 8-OHdG in aged rat lens. The amount of 8-OHdG in DNA was quantitatively measured by 8-OHdG ELISA. The level of 8-OHdG in DNA was significantly increased at 16 months (p<0.001, n=8) and at 26 months (p<0.001, n=8), compared with 2 month group. There was a significantly increase at 26 months (p<0.01, n=8) compared to 16 months group.

We compared young and aged lens tissues for the presence of 8-OHdG as an indicator of oxidative DNA damage. This approach can provide a quantitative estimate of oxidative DNA damage. As shown in Figure 3, the amount of 8-OHdG in DNA was significantly increased at 16 months (5.12±0.51, p<0.001, n=8) and at 26 months (6.27±0.66, p<0.001, n=8), compared with 2 month group (3.96±0.39, n=8). There was a significantly increase at 26 months compared to 16 months group (p<0.01, n=8). These data suggested that oxidative DNA damage increased in lenses with aging.

### Real time reverse transcriptase–polymerase chain reaction

**Figure 4:**
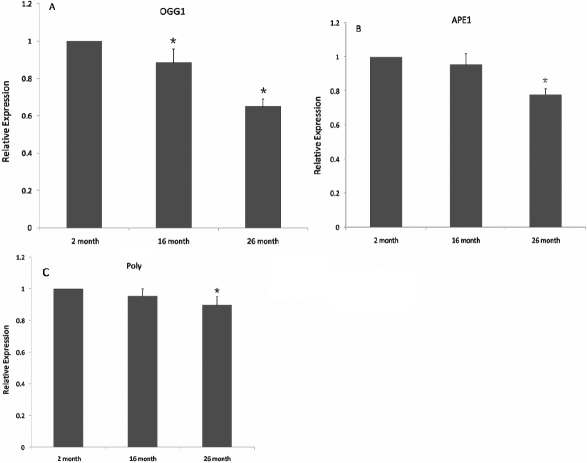
mRNA levels of BER enzymes are decreased in aged rat lens. Comparisons of the mRNA levels of BER enzymes: OGG1 (**A**), APE1 (**B**), and Polγ (**C**). GAPDH was used as internal standard for normalization. **A**: the expression level of OGG1 was decreased at 16 months (0.92±0.03, p<0.001, n=6) and 26 months (0.78±0.02, p<0.001, n=6), compared to the 2 month old group, and there was significantly decreased at 26 months (0.78±0.02, p<0.001, n=6) compared to 16 months group (0.92±0.03, n=6). **B**: it is shown that the expression level of APE1 was decreased at 26 months (0.78±0.03,p<0.001, n=6) as compared to the 16 month (0.95±0.06, n=6) and 2 month old group, and there was no significant change between 16 month (0.95±0.06, p=0.075,n=6) and 2 month old group. **C**: the expression level of Polγ was decreased at 26 months (0.90±0.05, p<0.001, n=6) compared to the 2 month old group, and there was a significantly decrease at 26 months (0.90±0.05, p=0.03, n=6) compared to the 16 months group (0.95±0.04, n=6).

The BER pathway is the predominant pathway for repairing oxidative mtDNA and nDNA damage. The components of BER were examined to determine their role in the repair of mtDNA and nDNA lesions in the present study. We chose to characterize enzymes OGG1, APE1, and Polγ at mRNA and protein levels, as they have a fundamental role in BER efficiency and cellular oxidative stress sensitivity. As shown in Figure 4A, the expression level of *OGG1* was decreased at 16 months (0.92±0.03, p<0.001, n=6) and 26 months (0.78±0.02, p<0.001, n=6), compared to the 2 month old group, and there was decreased at 26 months (0.78±0.02, p<0.001, n=6) compared to 16 months group (0.92±0.03, n=6). In Figure 4B, it is shown that the expression level of *APE1* was decreased at 26 months (0.78±0.03,p<0.001, n=6) as compared to the 16 month (0.95±0.06, n=6) and 2 month old group, and Figure 4C shows that the expression level of *Polγ* was decreased at 26 months (0.90±0.05, p<0.001, n=6) compared to the 2 month old group. The expression level of *Polγ* was decreased at 26 months (0.90±0.05, p=0.03, n=6) compared to 16 months group (0.95±0.04, n=6).

### Protein levels of BER DNA repair enzymes

**Figure 5:**
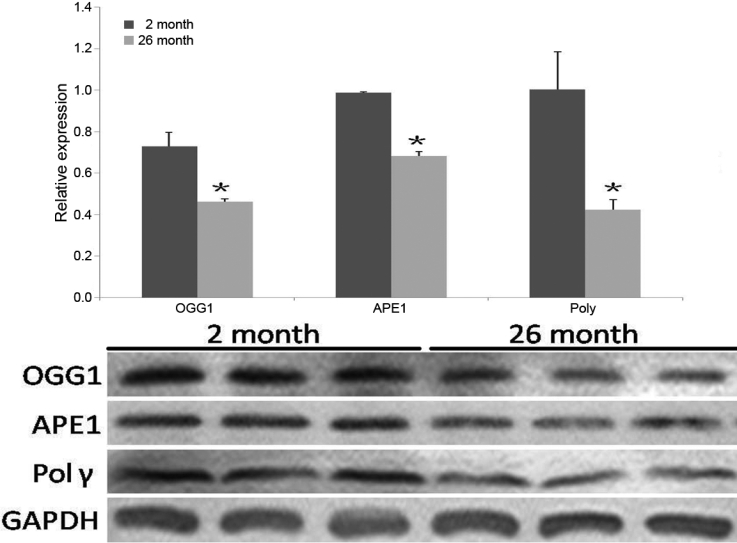
Protein levels of BER enzymes are decreased in aged rat lens. There were significant decreased in OGG1, APE1, and Polγ protein levels (0.46±0.01, p=0.003, n=3; 0.68±0.02, p=0.009, n=3;0.42±0.05, p=0.006, n=3,respectively) at 26 months when comparing to the 2 month old group (0.73±0.07, n=3; 0.99±0.004, n=3 and 1.00±0.18, n=3, respectively).

Protein levels of the enzymes of BER pathway in lenses were determined using antibodies against OGG1, APE1, and Polγ. As shown in Figure 5, there were significant decreased in OGG1, APE1, and Polγ protein levels (0.46±0.01, p=0.003, n=3; 0.68±0.02, p=0.009, n=3; 0.42±0.05, p=0.006, n=3,respectively) at 26 months when comparing to the 2 month old group (0.73±0.07, n=3; 0.99±0.004, n=3 and 1.00±0.18, n=3, respectively). Each band is representative of the lenses obtained from 3 rats.

### Immunohistochemistry

**Figure 6:**
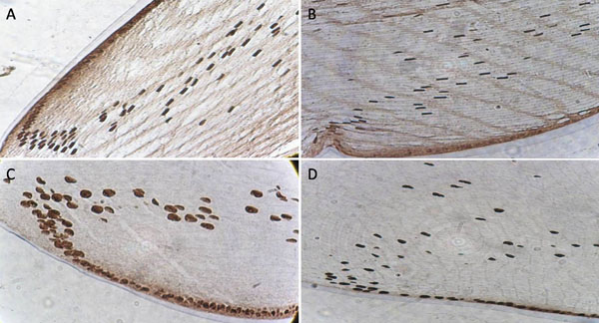
Immunohistochemical localization of OGG1 and APE1. Sectioned equals 7 μm thickness. OGG1 and APE1 were localized by immunohistochemistry within LECs and superficial fiber cells. **A**: Immunohistochemical localization of OGG1 in 2 months old rat lens. **B**: Immunohistochemical localization of OGG1 in 26 months old rat lens. **C**: Immunohistochemical localization of APE1 in 2 months old lens. **D**: Immunohistochemical localization of APE1 in 26 months old lens.

Immunohistochemistry revealed widespread immunoreactivity for OGG1 and APE1 in the lens. OGG1 and APE1 were localized by immunohistochemistry within LECs and superficial fiber cells (Figure 6). In Figure 6A, immunohistochemical localization of OGG1 in 2 months old is shown. Figure 6B shows the immunohistochemical localization of OGG1 in 26 month olds. Figure 6C demonstrates the immunohistochemical localization of APE1 in 2 month olds, while Figure 6D presents the same information for the 26 month old group. In the immunohistochemistry results, there was seemingly a decrease in the amount of fiber cells in aged rat lenses. The localization is predominantly in the nuclei. There is some reactivity in the cell cytoplasm; however, this is mostly restricted to the lens epithelium.

## Discussion

Cellular damage caused by the accumulation of mtDNA damage has been implicated in many disease processes, especially in age-related disorders, such as glaucoma and age-related macular degeneration (AMD). Mitochondria are significant endogenous sources of ROS. Mitochondria are located in the lens epithelium, the superficial fiber cells [3-5], and consume 90% of the oxygen entering the lens [5]. It is estimated that up to 4% to 5% of the consumed mitochondrial oxygen is converted to ROS [26,27]. It has been proposed that one of major function of the mitochondria in the lens is to maintain lens clarity by consuming oxygen and keeping the oxygen content at a very low level, preventing proteins and lipids from being oxidized [28]. Recent studies indicated that in mitochondria, ROS accumulates in subcapsular and cortical inclusions that colocalize with age-related cataract in old mouse lenses [29].

Our study is the first to examine mtDNA and nDNA damage in the lens in vivo with QPCR assays that combine with a new double strand DNA (dsDNA) reagent. This method is a gene and genome-specific QPCR assay that is able to detect mtDNA and nDNA damage and to allow the study of DNA repair [20]. This assay has been used in many in vivo and in vitro models and has been especially valuable in identifying oxidative stress-induced mtDNA damage [30]. EvaGreen is a newly developed DNA-binding dye that has been recently used in RT–PCR. It is stable both under PCR condition and during routine storage and handling. EvaGreen had a good binding affinity for dsDNA and low or no affinity for single strand DNA (ssDNA) [21-24]. Therefore, we replaced PicoGreen and SYBR Green with EvaGreen to perform QPCR and RT–PCR in this work.

In this study, there was an increase in oxidative mtDNA damage and mtDNA deletions as well as 8-OHdG, which is a product of oxidative DNA damage and is a sensitive marker of increased oxidative stress [31-33]. The increase of 8-OHdG reflected the increase of DNA damage with aging. Whereas we did not distinguish between nDNA and mtDNA as the source of increased 8-OHdG levels, our results implied the possibility that mtDNA contributes to the 8-OHdG signal because there was no increase nDNA damage in aged rat lens.

Oxidative mtDNA damage and mtDNA deletions have been considered important promoter in the normal process of aging [25]. However, these kinds of DNA damage are predominantly corrected by the BER pathway [30,34]. We chose three important BER enzymes: OGG1, APE1, and Polγ [30,35] to exam mtDNA and nDNA damage. We proved that OGG1 and APE1 were localized by immunohistochemistry within LECs, superficial fiber cells, and we provided further evidence to support the existence of a functional BER pathway in lens. In mammals, OGG1 is responsible for the removal of 8-oxoguanine, which arises through the incorporation, during DNA replication, by forming of 8-oxo- dGTP from oxidation of dGTP by ROS. APE1, by its turn, is an AP endonuclease that bypasses the AP lyase activity of OGG1, enhancing OGG1 turnover and producing a nick in the DNA backbone that allows for further processing to repair the DNA [36,37]. Polγ participates in all mtDNA metabolic processes and plays a gap filling role during excision repair, which is related to the final step of mtDNA repair or renewal. Unlike other mtDNA repair proteins, DNA polymerase γ is not a splice variant of a nuclear protein but is unique to mitochondria [38,39].

Our experiments demonstrated that the gene expression of mRNA and protein in these key BER enzymes decreased with aging, which caused the decrease of the repairing capability of mtDNA and the accumulation of mtDNA damage. There was not a significant increase in nDNA damage in aged rat lenses, although the gene expression of mRNA and protein in these key BER enzymes decreased with aging. We suggest the following two reasons to explain why there was not a significant increase in nDNA damage. First, genomic DNA is thought to be less sensitive to oxidative stress induced damage and genomic DNA damage persists shorter compared to mtDNA [40]. Second, repair of mtDNA and nDNA in principle could be very similar, with their difference being that nDNA repair pathways involve a larger number of proteins [41].

The increased mtDNA damage and decreased expression of BER enzymes may cause a “vicious cycle” of oxidative stress that possibly contributes to the accumulation of mtDNA mutations and age-related cataract pathogenesis. Whether by oxidation or deletion, mtDNA damage reduces respiratory activity with concomitant increases in free radical production [25,42,43]. Many previous studies also proved that the accumulation of oxidative mtDNA damage and mtDNA deletions can result in imbalances in the electron transport chain and release of cytochrome c into the cytoplasm, resulting in increased ROS and further causing the apoptosis and death in cells [11,44-47]. Previous studies have noted that the young lenses have a relatively uniform covering of LECs, whereas the old lenses have a lower density of surface LECs with frequent gaps [48]. Oxidant-induced mtDNA damage, mitochondrial dysfunction, and apoptosis or death of LECs may contribute to the onset of cataract [10,11,49]. At normal conditions, LECs use several strategies to maintain ROS at low levels to protect lipids, proteins, and nucleic acids. These strategies include activation of the ROS scavenger enzymes such as catalase, glutathione peroxidase, superoxide dismutase, and DNA repair enzymes. However, there is a diminution of these ROS scavenger enzymes and decreased DNA repair capability, placing the lens at risk for oxidative damage and cataract [50,51].

Finally, we suggest that the accumulation of oxidative mtDNA lesions and decline in ability to repair mtDNA damage may result in the onset of LECs dysfunction, emphasizing the importance of DNA-repair enzymes as pharmacological targets to promote DNA repair. These results may provide a strategy to prevent or slow the progress of cataract.
